# Multiparametric MRI-based intratumoral and peritumoral radiomics for predicting the pathological differentiation of hepatocellular carcinoma

**DOI:** 10.1186/s13244-024-01623-w

**Published:** 2024-03-27

**Authors:** Hai-Feng Liu, Min Wang, Qing Wang, Yang Lu, Yu-Jie Lu, Ye Sheng, Fei Xing, Ji-Lei Zhang, Sheng-Nan Yu, Wei Xing

**Affiliations:** 1 Department of Radiology, Third Affiliated Hospital of Soochow University, Changzhou, Jiangsu 213000 China; 2 Department of Anesthesiology, The Second People’s Hospital of Changzhou, Affiliated Hospital of Nanjing Medical University, Changzhou, China; 3 Department of Interventional Radiology, Third Affiliated Hospital of Soochow University, Changzhou, Jiangsu 213000 China; 4 Department of Radiology, Nantong Third People’s Hospital, Nantong, Jiangsu China; 5 Bayer Healthcare, Shanghai, China

**Keywords:** Radiomics, Multiparametric MRI, Differentiation, Carcinoma (hepatocellular)

## Abstract

**Purpose:**

To explore the predictive potential of intratumoral and multiregion peritumoral radiomics features extracted from multiparametric MRI for predicting pathological differentiation in hepatocellular carcinoma (HCC) patients.

**Methods:**

A total of 265 patients with 277 HCCs (training cohort *n* = 193, validation cohort *n* = 84) who underwent preoperative MRI were retrospectively analyzed. The risk factors identified through stepwise regression analysis were utilized to construct a clinical model. Radiomics models based on MRI (arterial phase, portal venous phase, delayed phase) across various regions (entire tumor, Peri_5mm, Peri_10mm, Peri_20mm) were developed using the LASSO approach. The features obtained from the intratumoral region and the optimal peritumoral region were combined to design the IntraPeri fusion model. Model performance was assessed using the area under the curve (AUC).

**Results:**

Larger size, non-smooth margins, and mosaic architecture were risk factors for poorly differentiated HCC (pHCC). The clinical model achieved AUCs of 0.77 and 0.73 in the training and validation cohorts, respectively, while the intratumoral model achieved corresponding AUC values of 0.92 and 0.82. The Peri_10mm model demonstrated superior performance to the Peri_5mm and Peri_20mm models, with AUC values of 0.87 vs. 0.84 vs. 0.73 in the training cohort and 0.80 vs. 0.77 vs. 0.68 in the validation cohort, respectively. The IntraPeri model exhibited remarkable AUC values of 0.95 and 0.86 in predicting pHCC in the training and validation cohorts, respectively.

**Conclusions:**

Our study highlights the potential of a multiparametric MRI-based radiomic model that integrates intratumoral and peritumoral features as a tool for predicting HCC differentiation.

**Critical relevance statement:**

Both clinical and multiparametric MRI-based radiomic models, particularly the intratumoral radiomic model, are non-invasive tools for predicting HCC differentiation. Importantly, the IntraPeri fusion model exhibited remarkable predictiveness for individualized HCC differentiation.

**Key points:**

• Both the intratumoral radiomics model and clinical features were useful for predicting HCC differentiation.

• The Peri_10mm radiomics model demonstrated better diagnostic ability than other peritumoral region-based models.

• The IntraPeri radiomics fusion model outperformed the other models for predicting HCC differentiation.

**Graphical Abstract:**

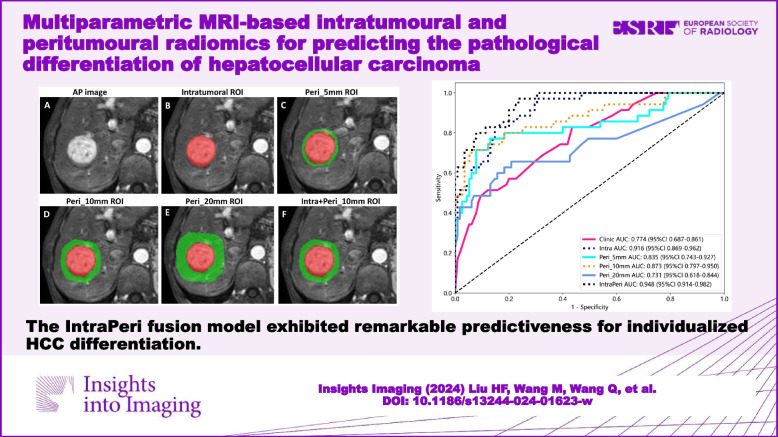

**Supplementary Information:**

The online version contains supplementary material available at 10.1186/s13244-024-01623-w.

## Introduction

Hepatocellular carcinoma (HCC) comprises 75–85% of all primary liver malignancies, making it the sixth most prevalent cancer worldwide and a leading cause of cancer-related mortality [1, 2]. The degree of tumor differentiation is a recognized indicator account for postoperative recurrence rates of 50–70% and poor survival outcomes in HCC patients [3, 4]. The preoperative prediction of HCC differentiation would offer significant benefits to patients, particularly those with good differentiation [5, 6], avoiding unnecessary chemotherapy, guiding preferred surgical resection methods, and achieving favorable outcomes. At present, liver biopsy serves as the primary method for preoperatively detecting HCC differentiation, but this method involves invasive procedures and potential sampling bias [7]. Therefore, there is an urgent demand to develop a precise and non-invasive imaging technique for predicting HCC differentiation.

Radiomics allows for thorough assessment of tumor heterogeneity at the microscopic scale by extracting numerous quantitative features from images and has shown significant capability in evaluating HCC characteristics [8]. Magnetic resonance imaging (MRI) is essential for non-invasively characterizing HCC, providing exceptional soft-tissue resolution and revealing the heterogeneity of HCC more accurately than other imaging techniques [8, 9]. Currently, several MRI-based radiomic studies have demonstrated enhanced predictive ability for determining HCC differentiation [10–14]. However, these studies have exclusively concentrated on intratumoral features, despite mounting evidence indicating that peritumoral features also offer complementary information. Recent research has highlighted the value of MRI-based peritumoral radiomic models for predicting microvascular invasion (MVI) [15], treatment response [16], and early recurrence [17] after resection or ablation in HCC patients. Furthermore, both Hu et al. [15] and Cao et al. [18] demonstrated that a combination of intratumoral and peritumoral features from MRI could more accurately predict MVI in HCC. These studies indicate that peritumoral features could provide valuable information in radiomic analysis.

However, a systematic exploration and comparison of MRI-based peritumoral radiomic models for predicting HCC differentiation is lacking, so the value of peritumoral features in predicting HCC differentiation remains controversial. Our study aimed to explore the performance of multiparametric MRI-based intratumoral and multiregional peritumoral radiomic models for predicting HCC differentiation. Furthermore, a combined IntraPeri radiomic model integrating both intratumoral and optimal peritumoral features was established and validated to achieve greater accuracy in predicting HCC differentiation.

## Materials and methods

### Patient selection

This study received approval from our Hospital Ethics Committee (2022-CL027-01), and the requirement for informed consent from the enrolled patients was waived. Between January 2017 and July 2023, 287 consecutive HCC patients who underwent preoperative MRI and subsequently received a diagnosis of HCC following hepatectomy were retrospectively analyzed. The exclusion criteria are as follows: (1) transarterial chemoembolization (TACE) or radiofrequency ablation therapy before MRI scanning (*n* = 6); (2) poor MR image quality or incomplete MRI examinations (*n* = 7); and (3) the lack of a tumor differentiation report (*n* = 9). Ultimately, the study involved 265 patients who were randomly classified into either the training or validation cohort with a cross-validation approach at a ratio of 7:3.

### Pathological differentiation analysis

The tumor specimens were subjected to hematoxylin and eosin (HE) staining to determine the degree of HCC differentiation by a proficient pathologist with 12 years of experience who was blinded to the preoperative examinations. Based on the classification criteria [19], the tumors were categorized as well-, moderately, or poorly differentiated HCC (pHCC). When HCC tumors displayed various differentiation results, the predominant differentiation determined the final diagnosis. Notably, we classified both moderately and well-differentiated HCC as non-poorly differentiated HCC (npHCC).

### MRI protocol

A 3.0-T MRI scanner (Magnetom Verio, Siemens, Germany) was used to perform the MRI scans. The following sequences were conducted: (1) T1WI; (2) T2WI; (3) echo-planar diffusion-weighted imaging (DWI), *b* values = 0.800 s/mm^2^; and (4) contrast-enhanced MRI (CEMRI) with an injection of 0.2 mL/kg of Gd-DTPA (Magnevist, Bayer, Germany) at a rate of 1 mL/s for bolus tracking. Three-dimensional (3D) volumetric interpolated breath-hold examination techniques were employed to capture arterial phase (AP, 25–35 s), portal venous phase (PVP, 60–70 s), and delayed phase (DP, 180 s) images. The entire CEMRI procedure lasted approximately 3–4 min. Additional MRI sequence details are shown in Table S1.

### Data collection

The baseline data, encompassing patient age, sex, etiology, alpha-fetoprotein (AFP) level, alanine aminotransferase (ALT) level, aspartate aminotransferase (AST) level, total bilirubin (TB) level, prothrombin time (PT), albumin level, Child–Pugh grade, performance status (PS), Barcelona Clinical Liver Cancer (BCLC) stage, HCC number, and differentiation, were collected from the electronic medical record system.

According to the Liver Imaging Reporting and Data System (LI-RADS) criteria (Version 2018) [20], MRI features were evaluated by two radiologists with 7 (H.F.L., radiologist 1) and 12 years (Q.W., radiologist 2) of experience in liver imaging. Both radiologists were blinded to the pathological results, and any inconsistencies were resolved through discussion under the supervision of senior radiologists (W.X., radiologist 3). The following features were collected: (a) major features: non-rim arterial phase hyperenhancement (NAPHE), non-peripheral washout, and enhancing capsules; (b) particular auxiliary features: nodule-in-nodule, mosaic architecture, blood products or higher fat content in HCC; (c) auxiliary features favoring malignancy but not HCC specifically: mild-to-moderate T2 hyperintensity, DP hypointensity, iron sparing in the tumor, and corona enhancement; and (d) baseline features: HCC size, margin, and shape.

### Clinical model development

Univariate and multivariate logistic regression methods were subsequently used to determine risk factors for pHCC in the training cohort. These significant factors were then integrated with a logistic regression algorithm to develop a clinical model for identifying pHCC, which was further assessed in the validation cohort.

### Imaging preprocessing, ROI segmentation, and peritumoral region dilation

Prior to region of interest (ROI) delineation, the images were resampled at a voxel spacing of 1 × 1 × 1 mm^3^ and grayscale normalized to compensate for voxel spatial differences and maintain grayscale consistency, respectively. The 3D-ROIs encompassing the entire tumor were manually delineated along the border on each successive transverse slice of the AP, PVP, and DP images by radiologist 1 using the open-source software ITK-SNAP (version 3.6.0, www.itk-snap.org), and all manual delineations were verified by senior radiologist 2. Furthermore, the MR images of 30 HCCs were randomly chosen for resegmentation by both radiologists 1 week later to redraw the ROIs.

**Fig. 1:**
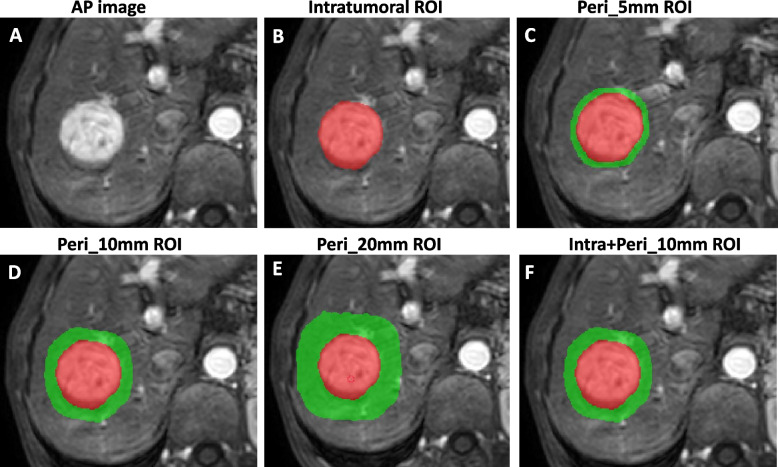
Examples of masks with different dilation distances on MR images. The red region represents the intratumoral region that was segmented by radiologists. The green-colored ring-like regions indicate the multiple peritumoral regions obtained with dilation

As the voxel spacing of the images was resampled to 1 × 1 × 1 mm^3^, the expanded voxel size was adjusted to the dilated peritumoral region by convolving the ROIs with a 3D box kernel. The peritumoral regions were then acquired with the SimpleITK package in Python software (version 3.6) by dilating the intratumoral 3D-ROIs by 5 mm, 10 mm, and 20 mm in 3D. Notably, areas beyond the liver parenchyma covered by the dilation were manually excluded. Figure 1 shows a representative example of an intratumoral mask and multiscale dilated peritumoral region.

### Radiomics feature extraction and dimension reduction

The PyRadiomics package (Version 3.6) was utilized to extract shape (*n* = 14), first-order (*n* = 198), texture analysis features (*n* = 803). Consequently, 1015 features were extracted from each MRI sequence (AP, PVP, and DP) for each region, resulting in the cumulative acquisition of 3045 features from the multiparametric MR images by combining the radiomic features from different regions, including the intratumoral, 5-mm peritumoral (Peri_5mm), 10-mm peritumoral (Peri_10mm), and 20-mm peritumoral (Peri_20mm) regions.

### Feature selection criteria and dimension reduction

A three-step procedure was sequentially used to select optimal features [21]. Initially, Spearman’s rank correlation was implemented to eliminate features with a correlation coefficient greater than 0.9. Next, tenfold cross-validation was applied in conjunction with the least absolute shrinkage and selection operator (LASSO) method to select the optimal features with non-zero coefficients, as determined by the optimal penalty parameter. Subsequently, the maximum relevance-minimum redundancy (mRMR) approach was performed to further reduce data dimensionality.

### Individual and fusion radiomics model development

The selected robust features were combined according to their respective coefficients to predict pHCC through the support vector machine (SVM) classifier, contributing to the development of individual radiomic models, including intratumoral, Peri_5mm, Peri_10mm, and Peri_20mm models. The prediction probabilities of each peritumoral model were compared to select the most effective model. Subsequently, the features selected from the optimal peritumoral region were integrated with the intratumoral features to design the IntraPeri fusion model.

### Statistical analysis

**Fig. 2:**
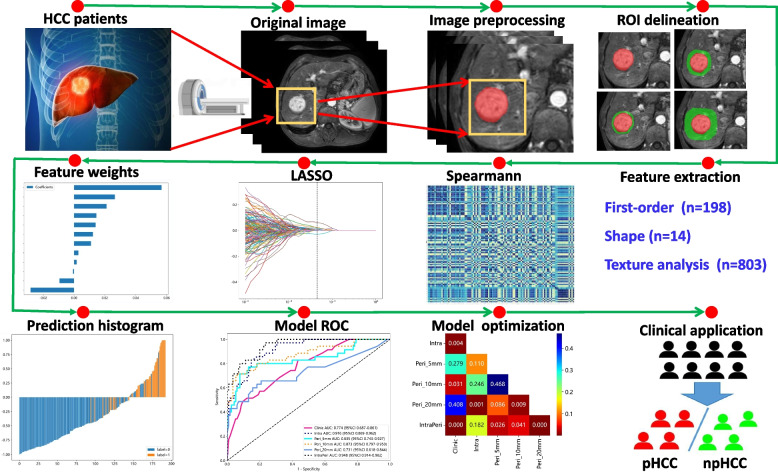
Detailed flowchart including MRI scanning, ROI segmentation and peritumoral region dilation, feature extraction and selection, and radiomic model construction and evaluation

Statistical analysis was performed with the R software (version 4.0). Categorical data are presented as percentages, while continuous variables are expressed as either the mean ± standard deviation or median (interquartile range) following normality testing using the Shapiro–Wilk method. The intraclass correlation coefficient (ICC) was utilized to analyze the variability of radiomic features between and within readers. The Mann–Whitney *U* or chi-square (*χ*^2^) test was employed to ascertain differences between pHCC and npHCC. The performance in predicting pHCC was assessed using the area under the receiver operating characteristic curve (AUC). Moreover, the DeLong method was employed to compare the differences in AUC values among the various models. A comprehensive flowchart illustrating the process from MRI scanning to model development is depicted in Fig. 2.

## Results

### Patient characteristics and HCC differentiation results

Two hundred and sixty-five patients with a total of 277 HCCs (eleven patients with two HCCs each) who underwent preoperative CEMRI were enrolled and randomly allocated into a training cohort (184 patients and 193 HCCs) and a validation cohort (81 patients and 84 HCCs). Among the 277 included HCCs, 48 (24.87%) and 145 (73.13%) in the training cohort and 26 (30.95%) and 58 (69.05%) in the validation cohort were diagnosed as pHCC and npHCC, respectively. The patient characteristics and HCC differentiation were comparable between training and validation cohorts, as shown in Table 1.

**Table 1:** Baseline characteristics of included patients diagnosed with HCC

	**Total** (*n* = 265)	**Training cohort** (*n* = 184)	**Validation cohort** (*n* = 81)	*p* value
**Age** (years)	62.80 ± 10.38	63.12 ± 10.18	62.09 ± 10.87	0.675
**Sex** (*n*, %)				
Male	210 (79.25)	145 (78.80)	66 (81.48)	0.618
Female	55 (20.75)	39 (21.20)	15 (81.48)	
**Etiology** (*n*, %)				
HBV/HCV	199 (75.09)	138 (75.00)	61 (75.31)	0.957
None	66 (24.91)	46 (25.00)	20 (24.69)	
**AFP** (*n*, %)				
≥ 20 ng/mL	107 (40.38)	78 (42.39)	29 (35.80)	0.314
< 20 ng/mL	158 (59.62)	106 (57.61)	52 (64.20)	
**ALT** (U/L)	26.80 (18.50–39.60)	26.85 (18.40–39.65)	26.20 (18.60–35.30)	0.715
**AST** (U/L)	31.75 (25.99–43.15)	31.80 (25.60–42.90)	31.70 (26.40–45.40)	0.814
**TB** (μmol/L)	13.60 (10.90–19.20)	13.25 (10.88–19.20)	15.00 (11.10–19.90)	0.253
**PT** (s)	12.20 (11.60–13.00)	12.30 (11.60–13.00)	12.10 (11.60–12.80)	0.438
**Albumin** (g/L)	40.31 (37.40–43.50)	40.20 (36.80–43.15)	40.80 (37.90–44.90)	0.134
**Child–Pugh grade** (*n*, %)				
A	237 (89.43)	166 (90.22)	71 (87.65)	0.532
B	28 (10.57)	18 (9.78)	10 (12.35)	
**PS** (*n*, %)				
0	262 (98.87)	182 (98.91)	80 (98.77)	0.917
1	3 (1.13)	2 (1.09)	1 (1.23)	
**HCC number** (*n*, %)				
Solitary	253 (95.47)	176 (95.65)	77 (95.06)	0.831
Two	12 (4.53)	8 (4.35)	4 (4.94)	
**BCLC stage** (*n*, %)				
0 ~ A	235 (88.68)	166 (90.22)	69 (85.19)	0.234
B ~ C	30 (11.32)	18 (9.78)	12 (14.81)	
**HCC differentiation** (*n*, %)				
Well/moderately	52 (11.4)/151 (53.85)	43 (22.27)/102 (54.40)	9 (15.51)/49 (58.33)	0.680
Poorly	74 (26.71)	48 (24.87)	26 (30.95)	

*HCC* hepatocellular carcinoma, *HBV* hepatitis B virus, *HCV* hepatitis C virus, *AFP* alpha-fetoprotein, *ALT* alanine aminotransferase, *AST* aspartate aminotransferase, *TB* total bilirubin, *PT* prothrombin time, *PS* performance status, *BCLC* Barcelona Clinical Liver Cancer. HCC differentiation rate was calculated based on HCC number (*n* = 277)

### Clinical risk features and model development

A larger tumor size, non-smooth margins, irregular shape, mosaic architecture, intratumoral blood products, intratumoral fat content, and corona enhancement were risk factors associated with pHCC (*p* < 0.05) in the training cohort, as shown in Table 2. After univariate and multivariate analysis, the remaining independent features associated with pHCC were larger tumor size, non-smooth margins, and mosaic architecture, as shown in Table 3. The clinical model, demonstrated AUC values of 0.77 (95% CI: 0.69–0.86) in the training cohort and 0.73 (95% CI: 0.45–1.00) in the validation cohort, respectively.

**Table 2:** Clinical features of each HCC nodule

**Clinical features**	**Training cohort** (*n* = 193)			**Validation cohort** (*n* = 84)		
	pHCC (*n* = 48)	npHCC (*n* = 145)	*p* value	pHCC (*n* = 26)	npHCC (*n* = 58)	*p* value
Tumor size (cm)	6.00 ± 3.95	4.15 ± 2.45	0.002	5.78 ± 3.44	4.63 ± 2.82	0.158
Non-smooth margin (*n*, %)	29 (60.42)	45 (31.03)	< 0.001	15 (57.69)	19 (32.76)	0.056
Irregular shape (*n*, %)	30 (62.50)	51 (35.17)	0.002	16 (61.54)	21 (36.21)	0.054
NAPHE (*n*, %)	47 (97.92)	140 (96.55)	1.0	26 (100.00)	57 (98.28)	1.0
Non-peripheral washout (*n*, %)	47 (97.92)	135 (93.10)	0.375	25 (96.15)	56 (96.55)	1.0
Enhancing capsule (*n*, %)	44 (91.67)	137 (94.48)	0.722	23 (88.46)	52 (89.66)	1.0
Nodule-in-nodule (*n*, %)	6 (12.50)	10 (6.90)	0.358	1 (3.85)	3 (5.17)	1.0
Mosaic architecture (*n*, %)	27 (56.25)	43 (29.66)	0.002	13 (50.00)	24 (41.38)	0.618
Intratumoral blood products (*n*, %)	21 (43.75)	35 (24.14)	0.016	10 (38.46)	15 (25.86)	0.363
Intratumoral fat content (*n*, %)	23 (47.92)	38 (26.21)	0.009	12 (46.15)	19 (32.76)	0.352
Mild-moderate T2 hyperintensity (*n*, %)	48 (100.00)	145 (100.00)	1.0	26 (100.00)	58 (100.00)	1.0
Corona enhancement (*n*, %)	26 (54.17)	41 (28.28)	0.002	13 (50.00)	22 (37.93)	0.425
Iron sparing (*n*, %)	41 (85.42)	134 (92.41)	0.247	23 (88.46)	53 (91.38)	0.985

*pHCC* poorly differentiated HCC, *npHCC* non-poorly differentiated HCC, *NAPHE* non-rim arterial phase hyperenhancement

**Table 3:** Univariate and multivariate analysis for pHCC

	**Univariate analysis**			**Multivariate analysis**		
**Variables**	OR	95% CI	*p* value	OR	95% CI	*p* value
Tumor size	1.04	1.02–1.05	< 0.001	1.12	1.10–1.14	0.002
Non-smooth margin	1.26	1.15–1.37	< 0.001	1.23	1.06–1.58	0.034
Irregular shape	1.24	1.14–1.35	< 0.001	1.03	0.86–1.23	0.791
NAPHE	1.14	0.86–1.50	0.453			
Non-peripheral washout	1.14	0.93–1.39	0.282			
Enhancing capsule	0.93	0.79–1.10	0.478			
Nodule-in-nodule	1.09	0.92–1.30	0.386			
Mosaic architecture	1.19	1.09–1.30	0.001	1.20	1.14–1.26	0.008
Intratumoral blood products	1.18	1.07–1.30	0.005	0.98	0.87–1.10	0.757
Intratumoral fat content	1.19	1.08–1.30	0.003	1.04	0.92–1.18	0.593
Mild-moderate T2 hyperintensity	0.0	0–∞	1.0			
Corona enhancement	1.20	1.10–1.31	0.001	0.93	0.79–1.10	0.49
Iron sparing	0.88	0.76–1.02	0.156			

### Individual radiomics model development

**Fig. 3:**
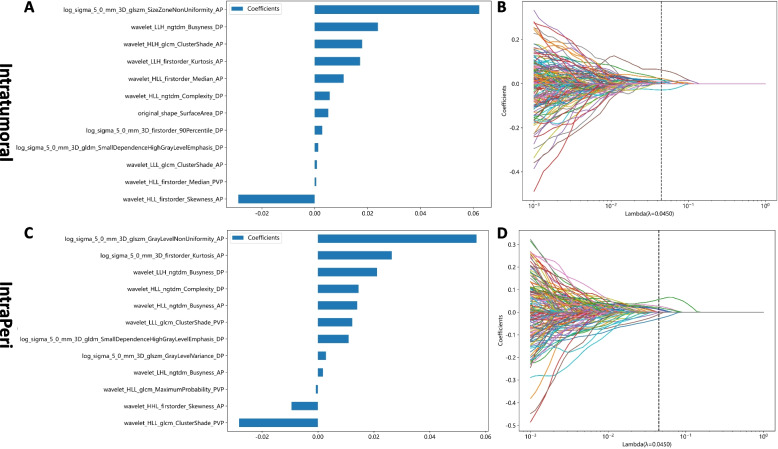
The weighted importances of the eighteen selected features using the LASSO approach in the intratumoral (**A**, **B**) and IntraPeri fusion models (**C**, **D**)

The inter- and intrareader variability for the intratumoral radiomics features was 0.88 (95% CI: 0.81–0.93) and 0.91 (95% CI: 0.84–0.95), respectively. After using the Spearman correlation method to eliminate redundant radiomic features, 319, 313, 318, and 291 features were retained from the intratumoral, Peri_5mm, Peri_10mm, and Peri_20mm regions, respectively. Following the mRMR and LASSO approaches, 12, 15, 7, and 7 highly robust features were retrained for training the intratumoral, Peri_5mm, Peri_10mm, and Peri_20mm models, respectively (Fig. 3, Figure S1-S8).

### Comparison of individual radiomics models

**Fig. 4:**
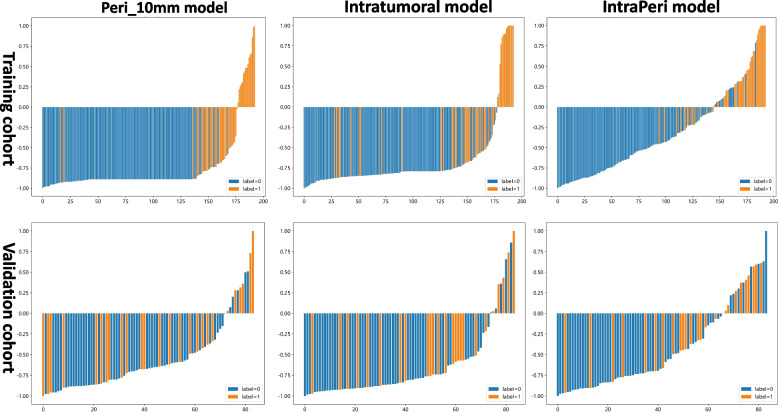
The ROC curves of the models (and the results of the DeLong test) in evaluating HCC differentiation in the training (**A**, **B**) and validation (**C**, **D**) cohorts

The AUCs of the different individual radiomics models in diagnosing pHCC ranged from 0.68 to 0.92 among all cohorts. The intratumoral model displayed an AUC value of 0.92 (95% CI: 0.87–0.96) and 0.82 (95% CI: 0.58–1.00). Among the three peritumoral models, the Peri_10mm model achieved the highest AUCs of 0.87 (95% CI: 0.80–0.95) and 0.80 (95% CI: 0.37–1.00), followed by the Peri_5mm model (AUCs = 0.84 and 0.77) and the Peri_20mm model (AUCs = 0.73 and 0.68) in the training and validation cohorts, respectively. The diagnostic ability of the different models to diagnose pHCC is provided in Table 4 and illustrated in Fig. 4.

**Table 4:** Diagnostic performance of the different model for predicting pHCC in the training and validation cohorts

Model name	Dilation distance (mm)	Number of features	Cohort	Accuracy	AUC (95% CI)	Sensitivity	Specificity
Clinical	NA	3	Training	0.63	0.77 (0.69–0.86)	0.83	0.57
			Validation	0.77	0.73 (0.45–1.00)	1.0	0.80
Intratumoral	NA	12	Training	0.85	0.92 (0.87–0.96)	0.83	0.85
			Validation	0.85	0.82 (0.58–1.00)	1.00	0.90
Peritumoral_5mm	5	15	Training	0.85	0.84 (0.74–0.93)	0.77	0.87
			Validation	0.77	0.77 (0.52–1.00)	1.00	0.80
Peritumoral_10mm	10	7	Training	0.89	0.87 (0.80–0.95)	0.71	0.94
			Validation	0.62	0.80 (0.37–1.00)	1.0	0.55
Peritumoral_20mm	20	7	Training	0.76	0.73 (0.62–0.84)	0.66	0.79
			Validation	0.62	0.68 (0.32–1.00)	1.00	0.60
IntraPeri	NA	12	Training	0.81	0.95 (0.91–0.98)	0.97	0.77
			Validation	0.77	0.86 (0.56–1.00)	1.00	0.73

### IntraPeri radiomics fusion model and DeLong test

**Fig. 5:**
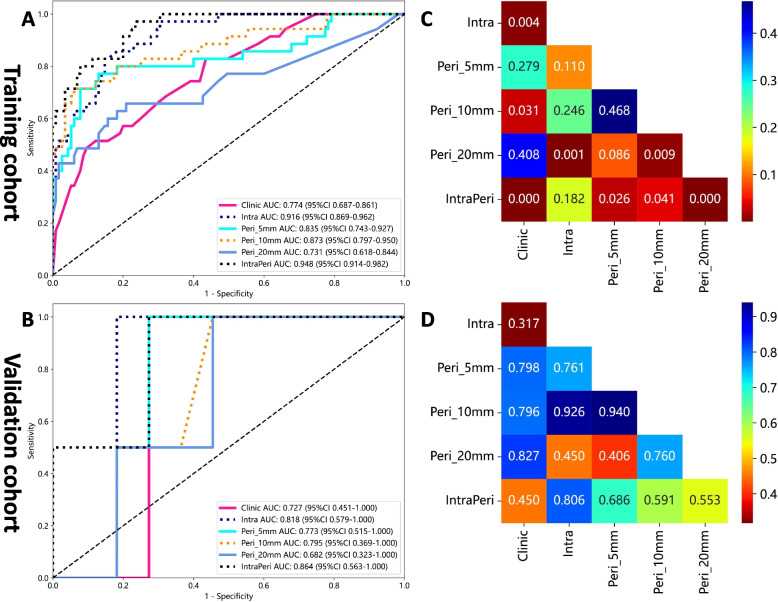
Sample prediction histograms of the intratumoral, Peri_10 mm, and IntraPeri radiomics models for predicting HCC differentiation in the training and validation cohorts. Label 1: pHCC; label 0: npHCC

By combining the features extracted from the intratumoral and Peri_10mm regions, the IntraPeri model, which integrated twelve significant features (Fig. 3), showed remarkable prediction efficacy, with AUCs = 0.95 (95% CI: 0.91–0.98) and 0.86 (95% CI: 0.56–1.00), respectively. The IntraPeri model had a greater AUC than did the intratumoral model and the clinical model (0.95 vs. 0.92 vs. 0.77 in the training cohort and 0.86 vs. 0.82 vs. 0.73 in the validation cohort). The sample prediction histogram of the corresponding model for predicting HCC differentiation is shown in Fig. 5.

## Discussion

Our study concluded that both the intratumoral and peritumoral models exhibited excellent performance in predicting HCC differentiation; notably, the intratumoral model demonstrated the best performance, with an AUC of 0.92. More meaningfully, the IntraPeri fusion model demonstrated the best performance. These findings indicate that combining intratumoral and peritumoral features provides an optimal alternative for predicting HCC differentiation.

Shinkawa et al. [3] found that tumor size is a risk factor associated with HCC recurrence following resection, which aligns with our findings, suggesting that increased tumor aggressiveness and infiltrative growth are associated with larger tumor sizes. A non-smoothed margin in a tumor is indicative of extranodular growth or the presence of multiple nodules. Moreover, a non-smooth margin can indicate destruction of the tumor capsule and invasion of the adjacent liver parenchyma, reflecting aggressive biological behavior. This finding reinforces our conclusion that non-smooth margins are significantly associated with pHCC, which is consistent with the findings of Liu et al [22]. Our study also revealed that a mosaic architecture is more common in pHCC, primarily due to its association with rapid disease progression and diverse biological heterogeneities [23]. Furthermore, the clinical model, which relies on visible MRI features, offers valuable performance for diagnosing pHCC and assisting radiologists in interpreting qualitative MRI features associated with pHCC.

Recently, Brancato et al. [14] and Yang et al. [12] developed CEMRI-based radiomics models for predicting HCC differentiation, but neither of the studies investigated peritumoral radiomics, and the inclusion of a relatively limited number of patients (*n* = 38–188) and the extraction of a restricted number of features (38–108 per sequence) were likely to decrease the reliability of the study findings, with AUC values ranging from 0.58 to 0.74. Our study concluded that the intratumoral model had a greater AUC for diagnosing pHCC than the clinical model; this can be attributed to the following factors: (1) radiomics, with its ability to provide a more thorough evaluation of tumor heterogeneity at the microscopic level by extracting numerous high-throughput features, is advantageous for accurately predicting pHCC which was identified based on a comprehensive assessment of heterogeneous pathologies [19]. (2) In contrast to clinical model that only relied on naked-eye features for qualitative evaluation, radiomics features served as quantitative imaging biomarkers for characterizing HCC biomedical behavior with better accuracy and more objective.

More importantly, we observed higher AUCs than did previous CEMRI-based intratumoral radiomic studies [12, 14]. This improvement can primarily be attributed to the inclusion of a large number (*n* = 3045) of features extracted from each HCC nodule in our study. It is worth noting that, to mitigate overfitting and multicollinearity resulting from the extensive number of features, we sequentially employed the Spearman rank correlation, LASSO, and mRMR approaches to reduce dimensionality and identify the optimal features, according to the checklist for conducting radiomics research [21]. Additionally, our study carefully selected twelve optimal features and combined them to construct the intratumoral model using an SVM classifier. This approach significantly contributed to the higher performance observed in diagnosing HCC differentiation. Furthermore, the intratumoral model exhibited higher AUC values compared to all peritumoral models. This can be attributed primarily to the fact that HCC differentiation is determined by intratumoral pathological features, such as the mitotic count, tumor differentiation, and presence of necrosis.

Previous studies have predominantly examined HCC without considering the peritumoral liver parenchyma in radiomics analysis [10–14]. Our results not only demonstrated the promising of the peritumoral radiomics model in predicting HCC differentiation but also indicated that the selection of the peritumoral region influences the prediction results of the radiomics pipeline. In this study, the Peri_10mm model presented the best performance in both cohorts, which confirms the results of a previous work showing that a radiomics model built from features in the peritumoral region 10 mm from the tumor border presented with higher accuracy in predicting MVI in HCC [24]. The peritumoral region [25, 26], known for its enrichment in edematous tissue, lymphatic or lymphocytic infiltration, and lymphangiogenesis, has been acknowledged as an integral part of HCC tissue. This region is thought to contribute to the heterogeneity observed in the extraction of marginal radiomics features. However, our study revealed that both the Peri_5mm and Peri_20mm models exhibited limited predictive ability for HCC differentiation. This can be attributed to the fact that a margin that is too narrow (< 5 mm) may lack sufficient information, whereas an excessively wide margin may introduce larger blood vessels and bile ducts, thus increasing the peritumoral heterogeneity and therefore decrease the predictive performance of peritumoral radiomics model.

Radiomics fusion models combining intratumoral and peritumoral features have emerged as highly effective decision for evidence-based management of patients with HCC. In a study conducted by Chong et al. [24], a radiomics fusion model was designed and demonstrated its potential as a biomarker for predicting MVI and stratifying the prognosis for solitary HCCs ≤ 5 cm. Additionally, Chen et al. [16] discovered that a radiomics fusion model showed improved AUC values in predicting the tumor response to TACE. The present research aimed to evaluate the value of our developed fusion model in evaluating HCC differentiation, achieving a high accuracy of 0.95. Our findings demonstrate the precision and discriminatory ability of our IntraPeri fusion model speculated that the complementary relationship between intratumoral and peritumoral features can provide more accurate information, thus highlighting its potential in improving individualized clinical diagnosis of HCC differentiation.

This study has several limitations that require consideration. First, this was a retrospective study that excluded suspected HCC patients without a suitable pathology and lacked external validation, potentially introducing selection bias and undermining the reliability of our study’s conclusions. Further validation through prospective studies is necessary to ensure the clinical applicability of these findings in our study. Second, it should be noted that the radiomics features were only extracted from CEMRI images, potentially overlooking significant features in DWI or T2WI images [9]. While CEMRI characteristics are known for representing the heterogeneity of HCC, the incorporation of additional imaging modalities could lead to a more comprehensive analysis in future studies. Third, these LI-RADS features are liable to suffer from personal bias resulting from radiologist interpretation, thus potentially introducing interobserver variability and subjectivity. This study did not evaluate the consistency of the researchers’ results, not only because previous research has reported high consistency among LI-RADS but also because comparisons of many features would deviate from the focus of our study on imaging biomarkers. Fourth, the peritumoral region was automatically expanded based on the intratumoral region, though areas beyond the liver parenchyma covered in the dilation were manually excluded from our study. However, in HCC patients with larger tumor volumes, the choice of optimal peritumoral region (10 mm) may not be generalizable, as the automatically dilated peritumoral region may not be heterogeneous in the liver. Ultimately, this study concentrated exclusively on examining the association between HCC differentiation and radiomic features. Subsequent research should also address the correlation between radiomic features and HCC recurrence and prognosis to further enhance the clinical applicability of our results.

## Conclusion

The present study emphasizes the potential of both clinical and multiparametric MRI-based radiomic models, particularly the intratumoral model, as non-invasive tools for predicting HCC differentiation. More importantly, the IntraPeri model, which integrates intratumoral and optimal peritumoral features, exhibited outstanding ability to predict individualized HCC differentiation.

### Supplementary Information

**Supplementary Material 1.**

**Supplementary Material 2.**

## Data Availability

The datasets used and/or analyzed during the current study are available from the corresponding author upon reasonable request.

## Abbreviations

AUC: Area under the ROC curve
HBP: Hepatobiliary phase
HCC: Hepatocellular carcinoma
LASSO: Least absolute shrinkage and selection operator
LI-RADS: Liver imaging reporting and data system
MRI: Magnetic resonance imaging
mRMR: Maximum relevance minimum redundancy
npHCC: Non-poorly differentiated HCC
pHCC: Poorly differentiated HCC
ROC: Receiver operating characteristic curve
SVM: Support vector machine
