# Supplementary material for: Multiparametric MRI-based intratumoral and peritumoral radiomics for predicting the pathological differentiation of hepatocellular carcinoma
Source: Insights Imaging. 2024 Mar 27;15:97. doi: 10.1186/s13244-024-01623-w (PMC10973314; doi:10.1186/s13244-024-01623-w)
Supplement: Supplementary file 1 — Supplementary Material 1. [file 13244_2024_1623_MOESM1_ESM.doc]

| **Table S1. MRI Sequences and Parameters** | | | | | |
| --- | --- | --- | --- | --- | --- |
| Parameters | T1WI | T2WI | FS-T2WI | DWI | DCE-MRI |
| Sequence name | TSE | FSE | FSE | EPI | 3D-VIBE |
| Orientation | Axial | Axial | Axial | Axial | Axial |
| Breath technique | Free breath | Free breath | Free breath | Free breath | Free breath |
| TR/TE (ms) | 5.48/2.46 | 700/97 | 700/97 | 1700/75 | 3.92/1.39 |
| FOV (mm^2^) | 380 × 380 | 380 × 380 | 380 × 380 | 380 × 380 | 380 × 380 |
| Matrix | 156 × 156 | 174 × 132 | 116 × 115 | 152 × 150 | 256 × 192 |
| Slice thickness/gap (mm) | 4/1 | 4/1 | 4/1 | 6/1 | 2/1 |
| Flip angle (degrees) | 10° | 90° | 90° | 90° | 10° |
| Bandwidth (Hz/pixel) | 500 | 482 | 482 | 2442 | 400 |
| NEX | 1 | 1 | 1 | 1 | 1 |
| b value (s/mm2) | – | – | – | 0, 800 | – |
| Scanning time (s) | 14 | 23 | 23 | 37 | 68 |
| Note: T1WI = T1-weighted imaging; T2WI = T2-weighted imaging; FS = Fat suppressed; DWI = diffusion-weighted imaging, DCE = dynamic contrast-enhanced; TSE = turbo spin echo; FSE = fast spin echo; EPI = echo planar imaging; VIBE = volumetric interpolated breath-hold examination; TR = repetition time; TE = echo time; FOV = field of view. | | | | | |
