# Supplementary material for: Multiparametric MRI-based intratumoral and peritumoral radiomics for predicting the pathological differentiation of hepatocellular carcinoma
Source: Insights Imaging. 2024 Mar 27;15:97. doi: 10.1186/s13244-024-01623-w (PMC10973314; doi:10.1186/s13244-024-01623-w)
Supplement: Supplementary file 2 — Supplementary Material 2. [file 13244_2024_1623_MOESM2_ESM.doc]

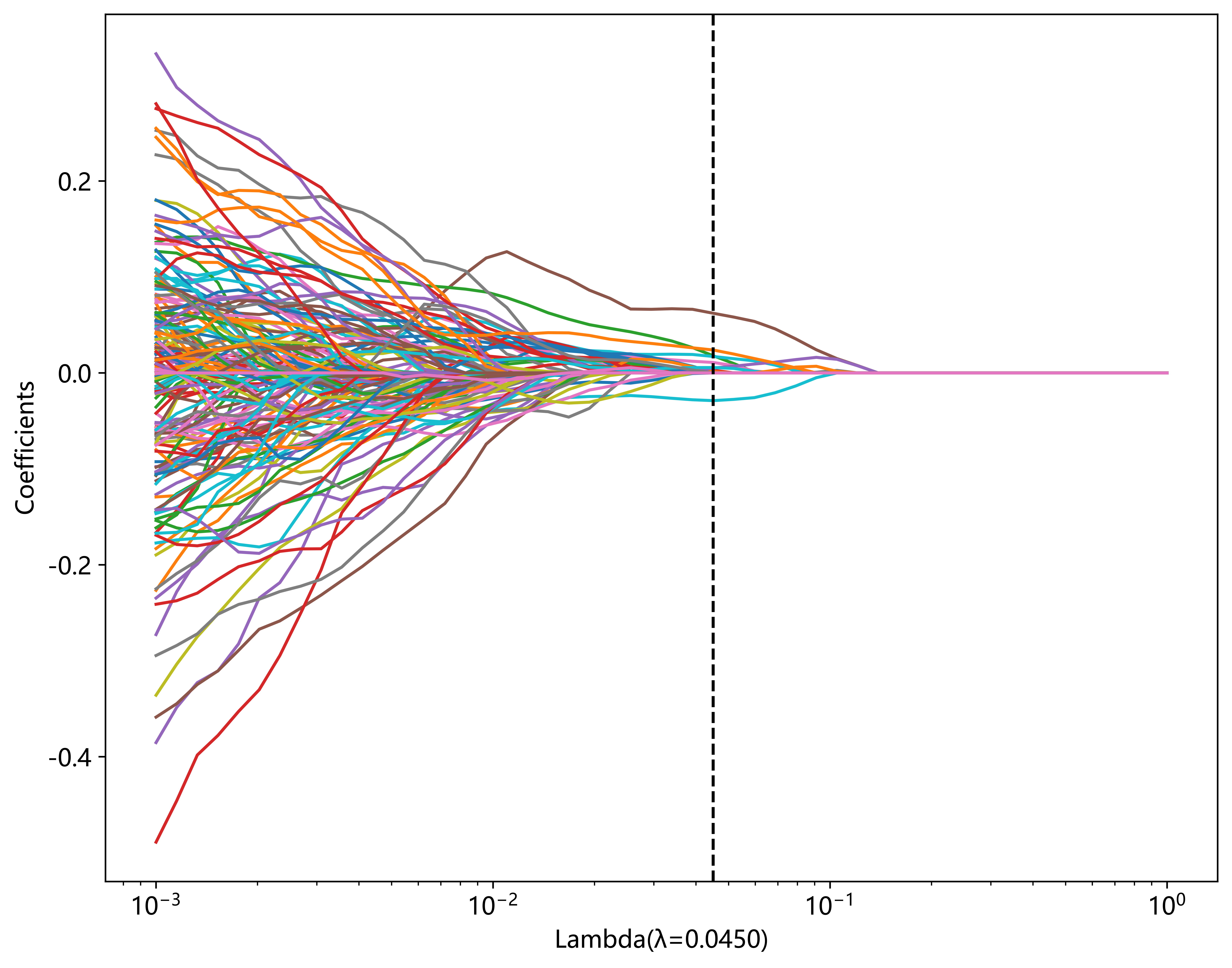


**Figure.S1 LASSO approach of intratumoral model**

**
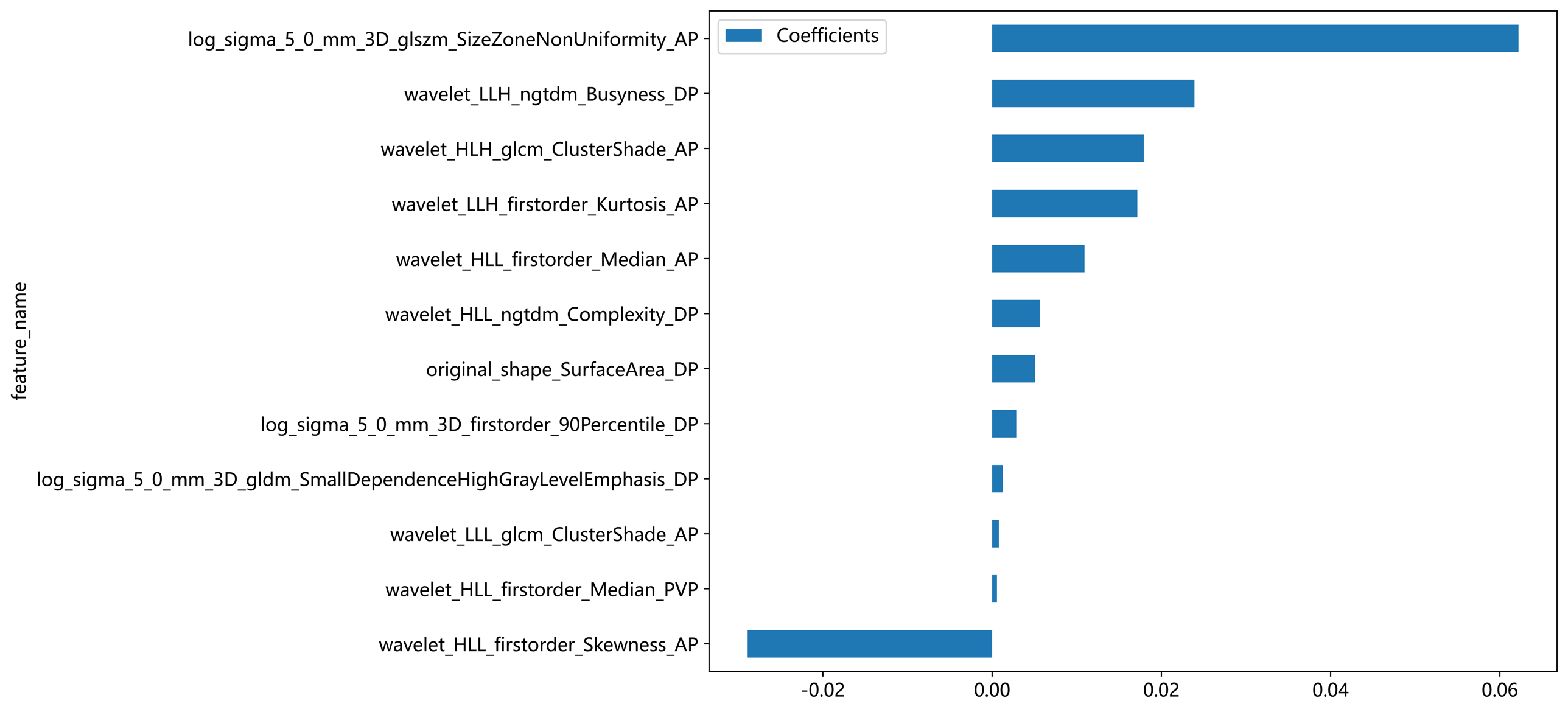
**

**Figure.S2** **Weighted importance of the 12 radiomics signatures of intratumoral model**

**
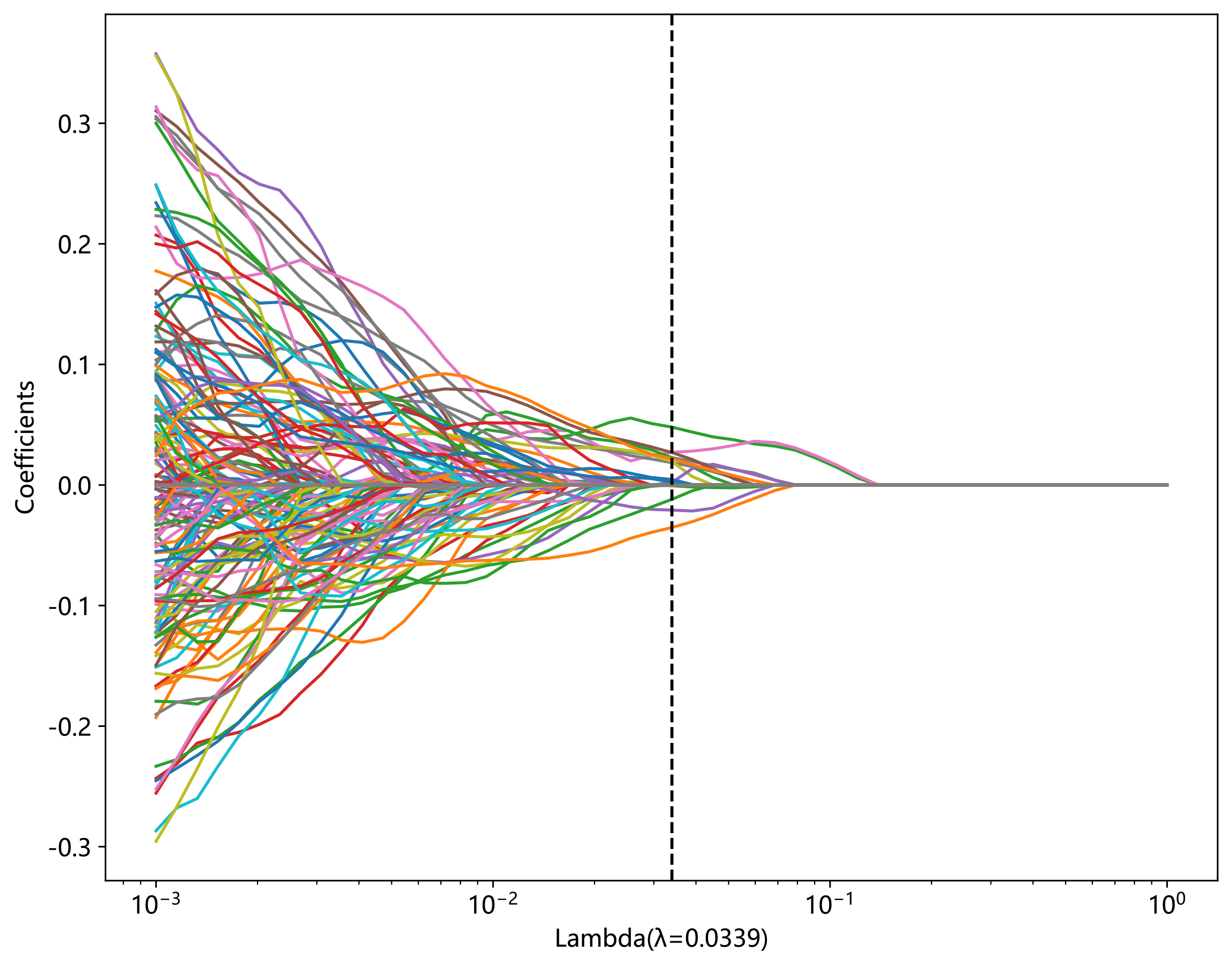
**

**Figure.S3 LASSO approach of Peri-5mm radiomics model**

**
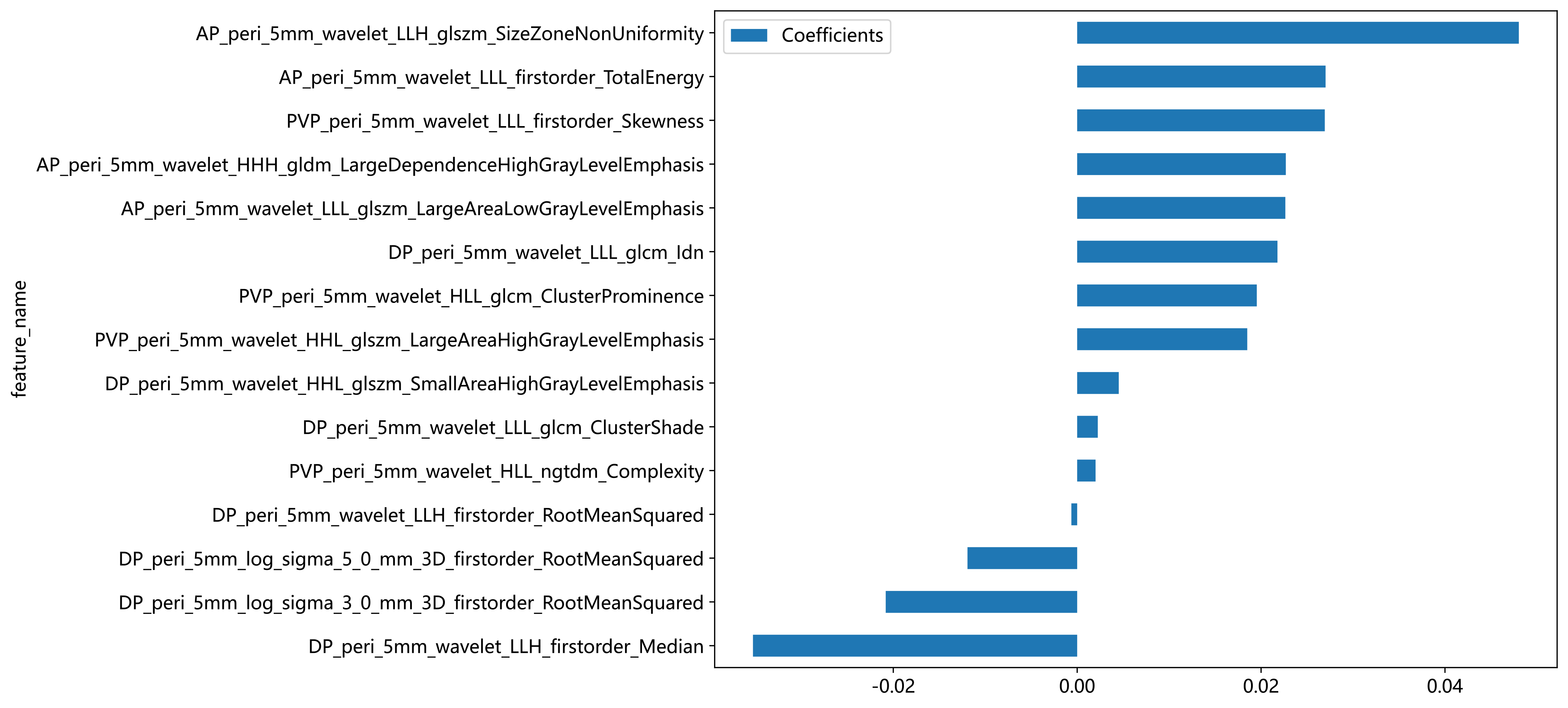
**

**Figure.S4** **Weighted importance of the 15 radiomics signatures of Peri-5mm model**

**
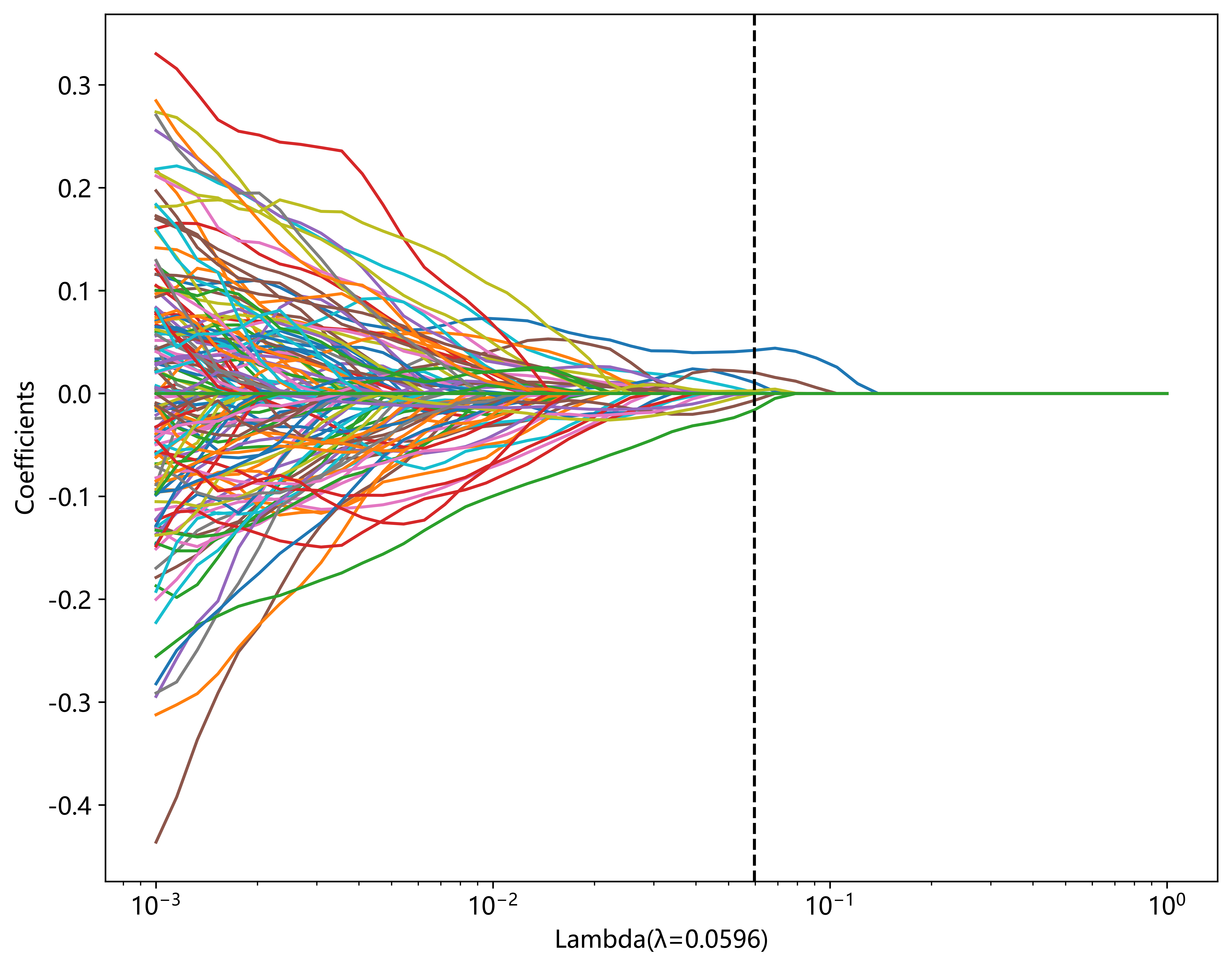
**

**Figure.S5 LASSO approach of Peri-10mm radiomics model**

**
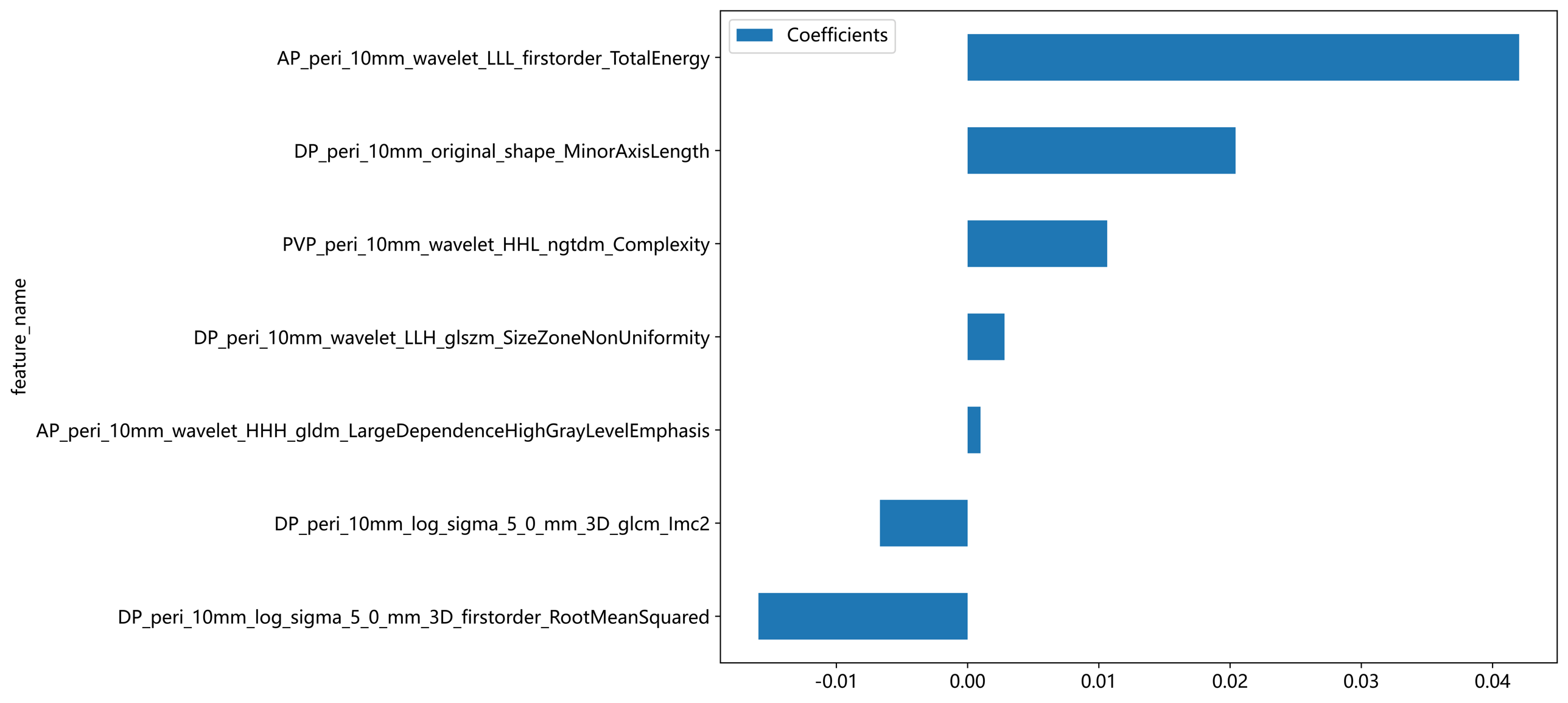
**

**Figure.S6** **Weighted importance of the selected 7 signatures of Peri-10mm model**

**
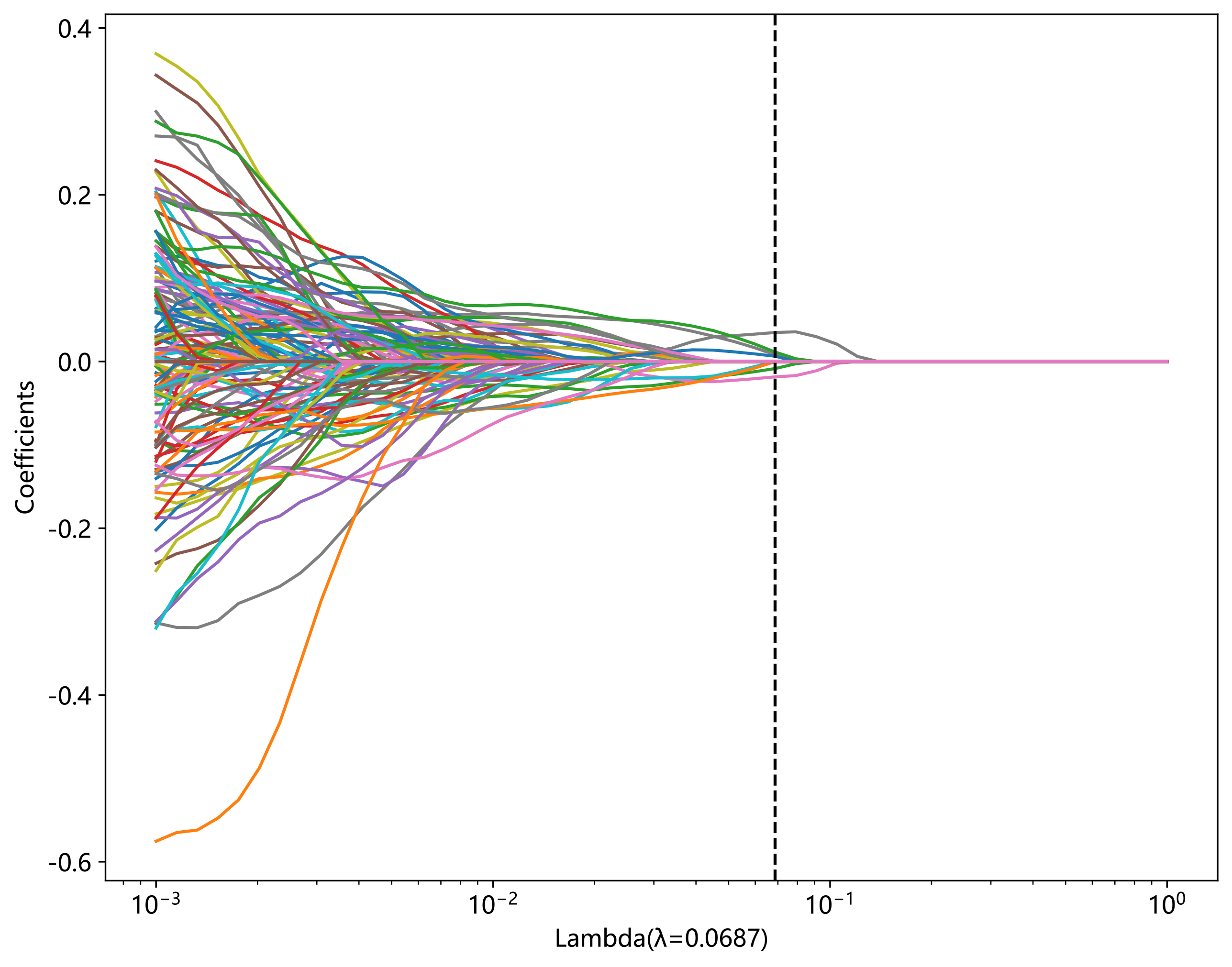
**

**Figure.S7 LASSO approach of Peri-20mm radiomics model**

**
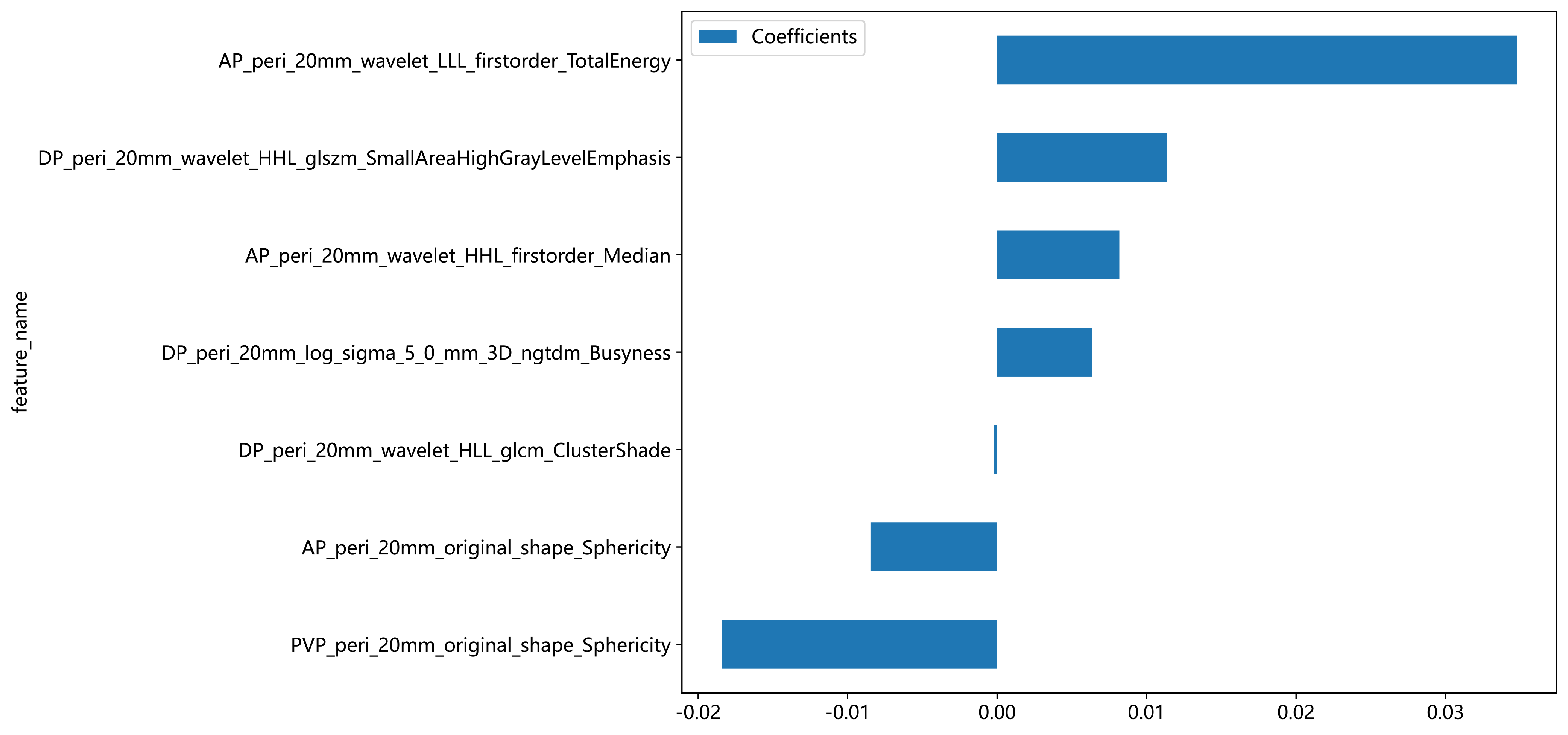
**

**Figure.S8** **Weighted importance of the selected 7 signatures of Peri-20mm model**
